# Supplementary material for: Structural Features of the Fragments from Cast Iron Cauldrons of the Medieval Golden Horde: Neutron Tomography Data
Source: J Imaging. 2023 May 11;9(5):97. doi: 10.3390/jimaging9050097 (PMC10218852; doi:10.3390/jimaging9050097)
Supplement: Supplementary file 1 [file jimaging-09-00097-s001.zip › jimaging-2345174-supplementary.pdf]

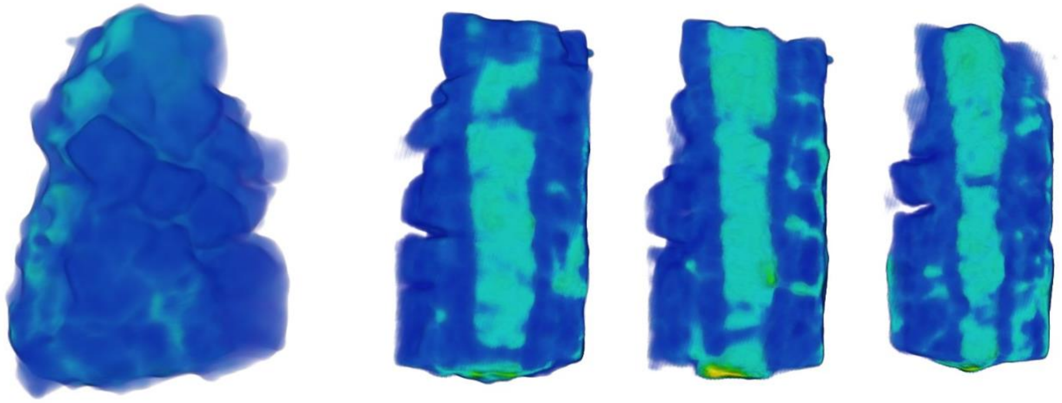

(a) Three-dimensional model of the sample BS-25 and several virtual slices. The color scheme corresponds to the effective attenuation coefficient of the neutron beam from weak (blue) to strong (red).

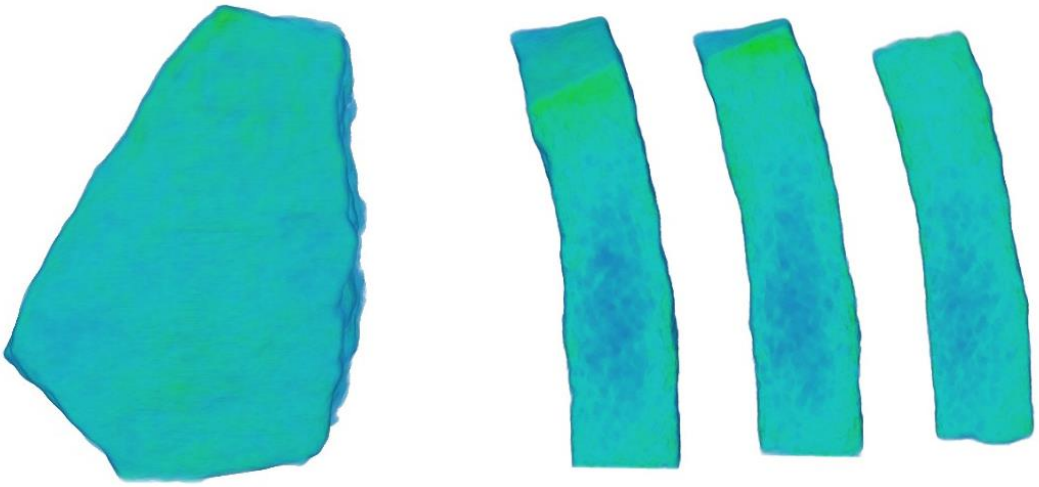

(b) Three-dimensional model of the sample BS-48 and several virtual slices. The color scheme corresponds to the effective attenuation coefficient of the neutron beam from weak (blue) to strong (red).

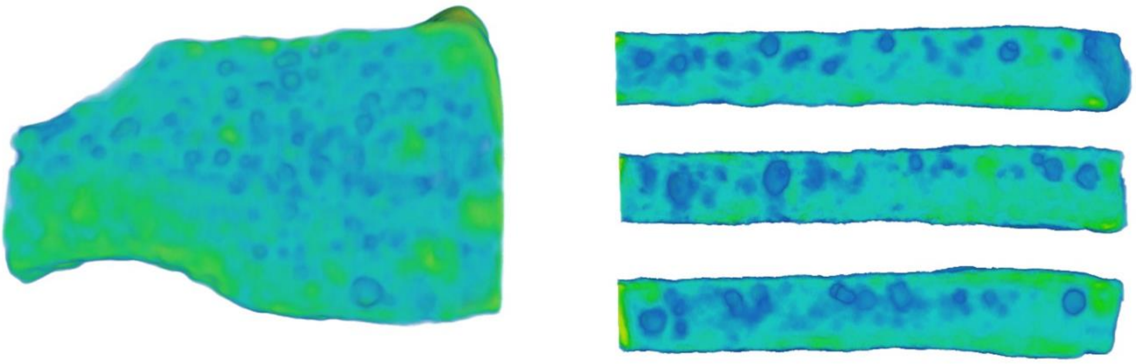

(c) Three-dimensional model of the sample BS-65 and several virtual slices. The color scheme corresponds to the effective attenuation coefficient of the neutron beam from weak (blue) to strong (red).

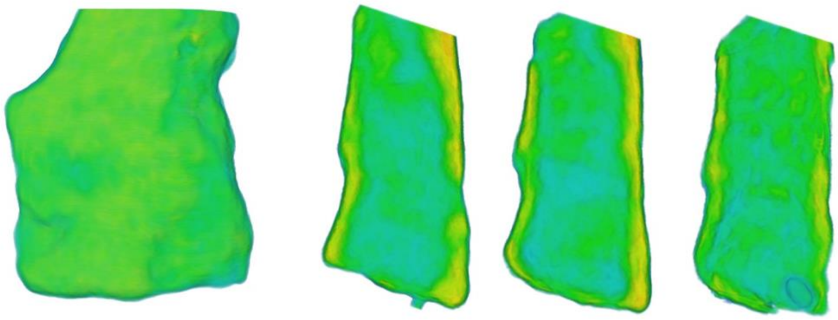

(d) Three-dimensional model of the sample BS-69 and several virtual slices. The color scheme corresponds to the effective attenuation coefficient of the neutron beam from weak (blue) to strong (red).

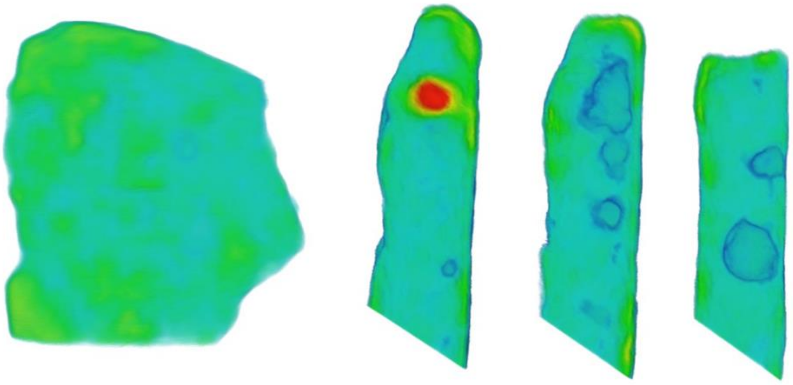

(e) Three-dimensional model of the sample BS-73 and several virtual slices. The color scheme corresponds to the effective attenuation coefficient of the neutron beam from weak (blue) to strong (red).

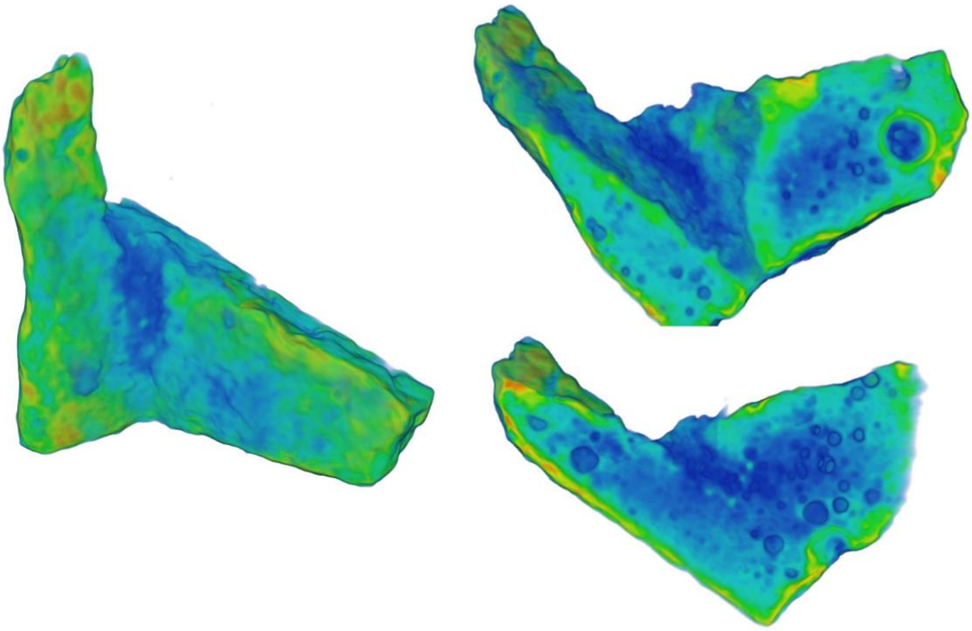

(f) Three-dimensional model of the sample SS-3 and several virtual slices. The color scheme corresponds to the effective attenuation coefficient of the neutron beam from weak (blue) to strong (red).

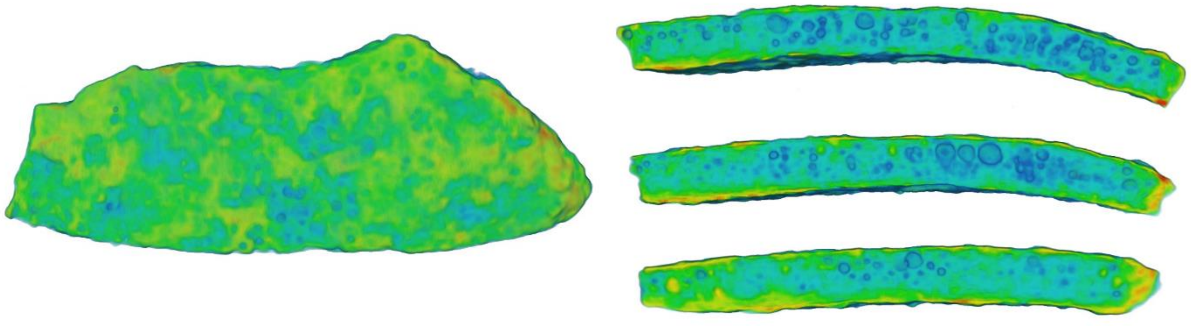

(g) Three-dimensional model of the sample SS-5 and several virtual slices. The color scheme corresponds to the effective attenuation coefficient of the neutron beam from weak (blue) to strong (red).

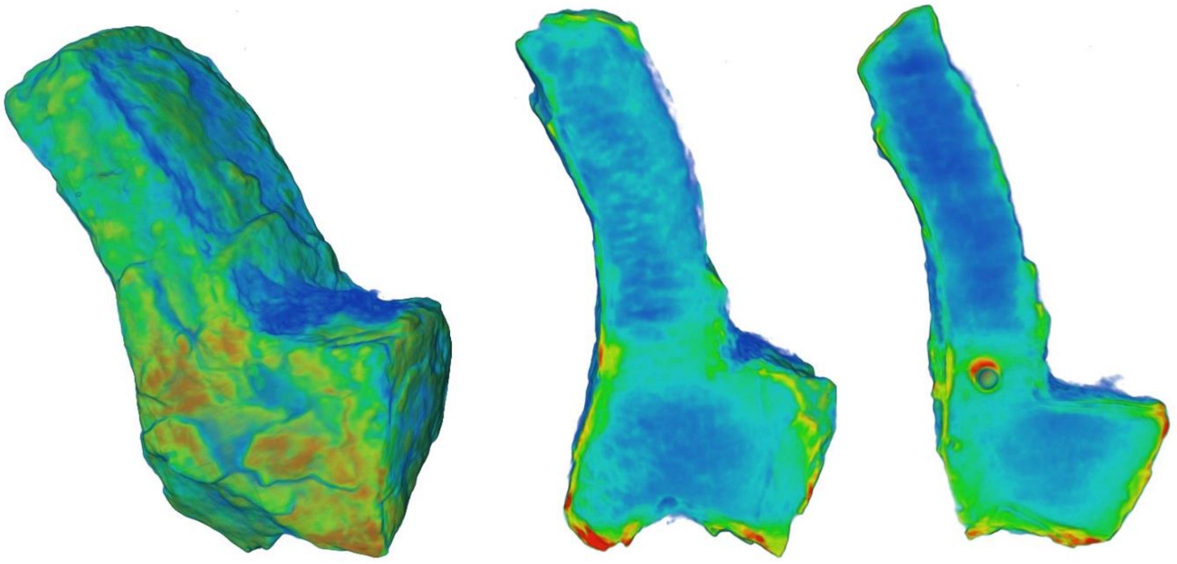

(h) Three-dimensional model of the sample SS-6 and several virtual slices. The color scheme corresponds to the effective attenuation coefficient of the neutron beam from weak (blue) to strong (red).

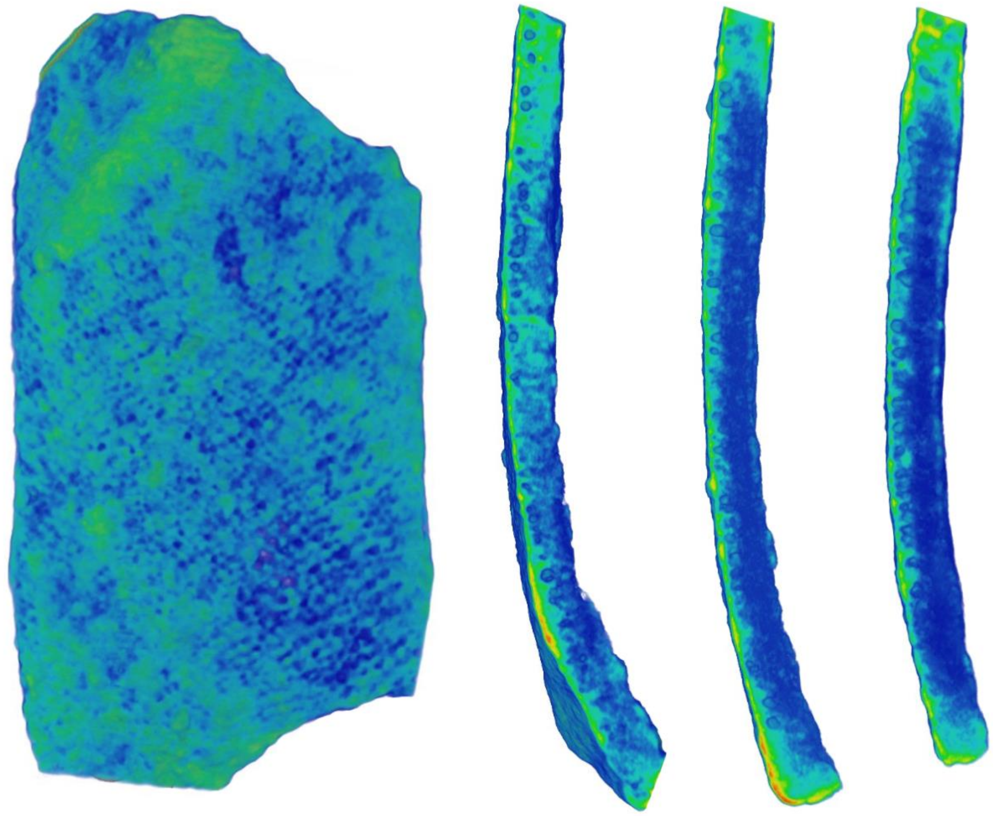

(I) Three-dimensional model of the sample SS-7 and several virtual slices. The color scheme corresponds to the effective attenuation coefficient of the neutron beam from weak (blue) to strong (red).

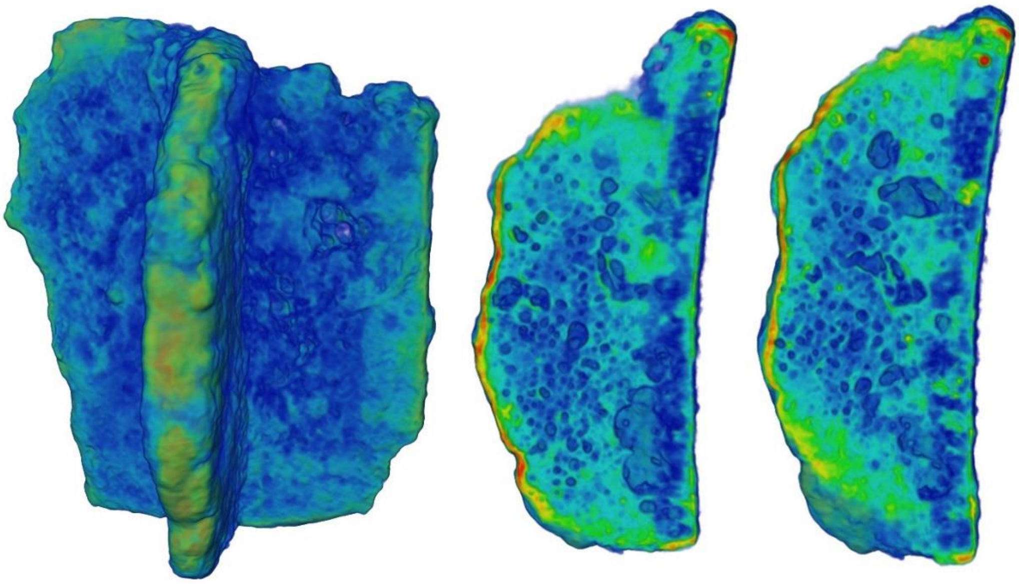

(i) Three-dimensional model of the sample SS-8 and several virtual slices. The color scheme corresponds to the effective attenuation coefficient of the neutron beam from weak (blue) to strong (red).

Figure S1. The reconstructed 3D model and some longitudinal and transverse slices of the cast iron fragments. Scale bars are presented.

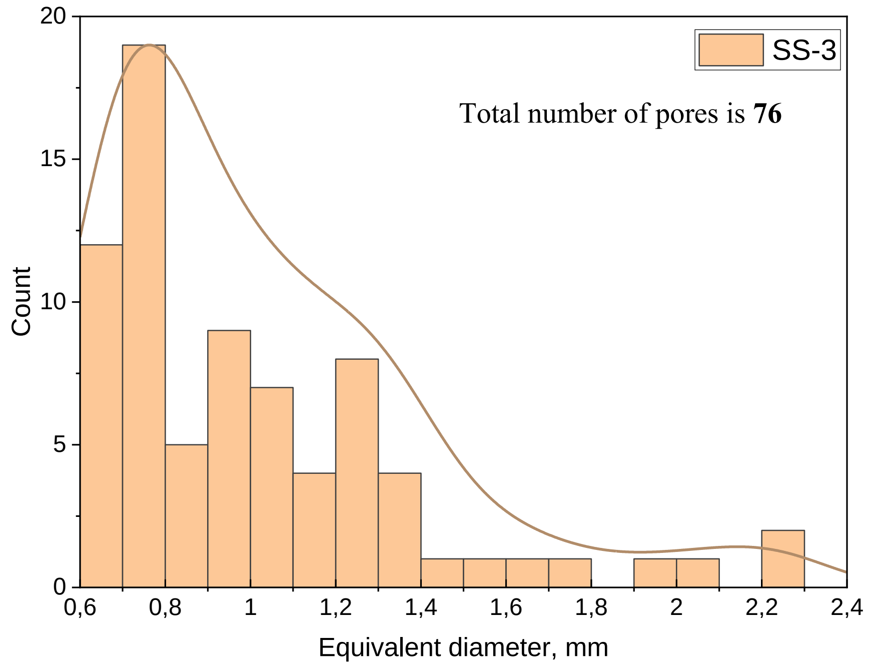

(a) The obtained distributions of the equivalent diameters of the pores with the Kernel smooth distribution curve for sample SS-3.

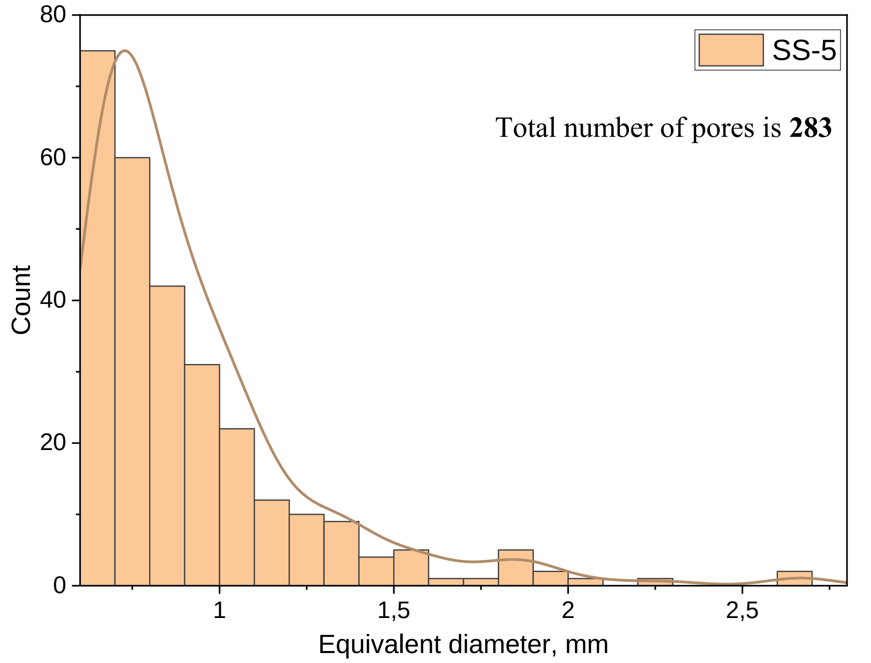

(b) The obtained distributions of the equivalent diameters of the pores with the Kernel smooth distribution curve for sample SS-5.

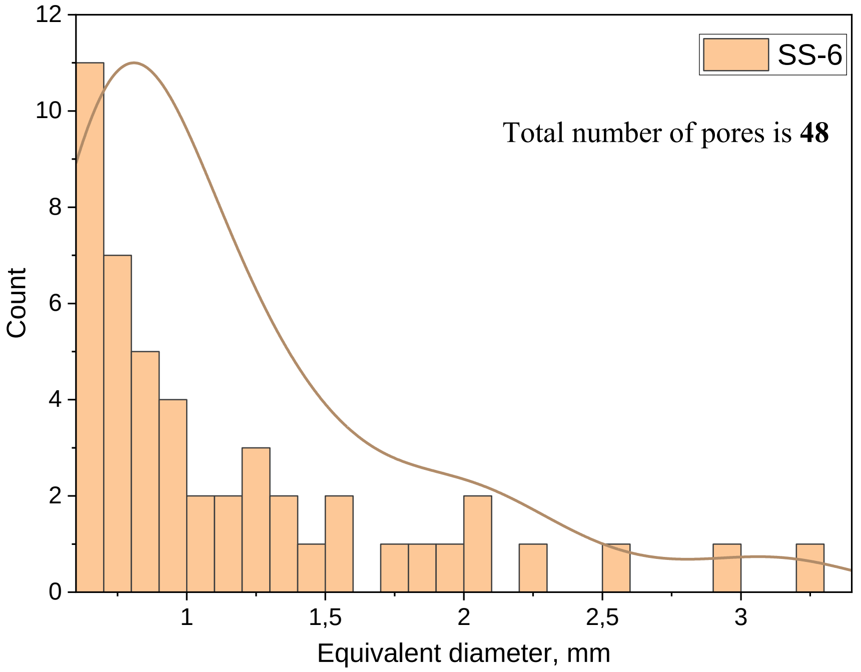

(c) The obtained distributions of the equivalent diameters of the pores with the Kernel smooth distribution curve for sample SS-6.

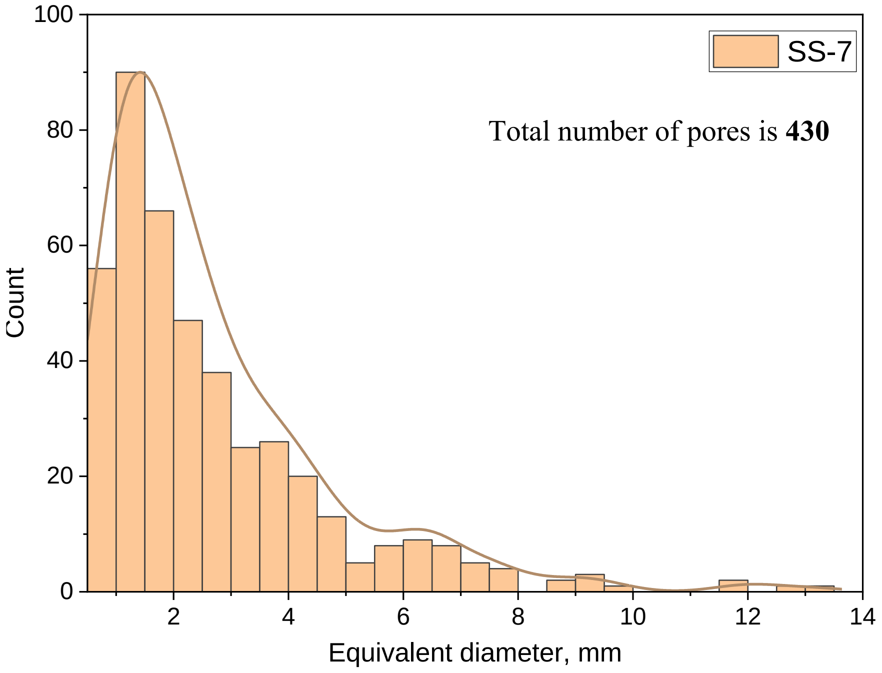

(d) The obtained distributions of the equivalent diameters of the pores with the Kernel smooth distribution curve for sample SS-7.

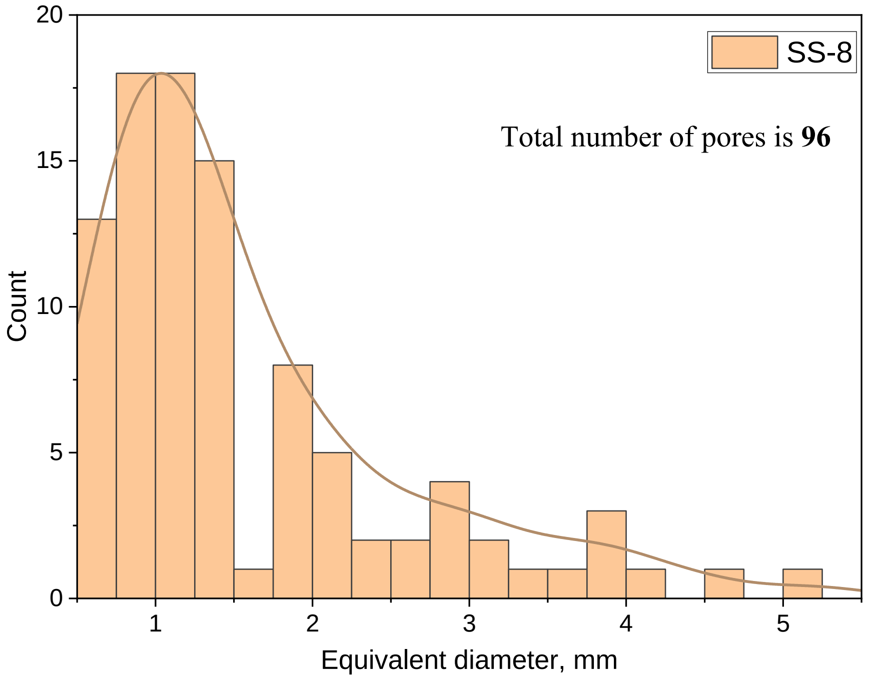

(e) The obtained distributions of the equivalent diameters of the pores with the Kernel smooth distribution curve for sample SS-8.

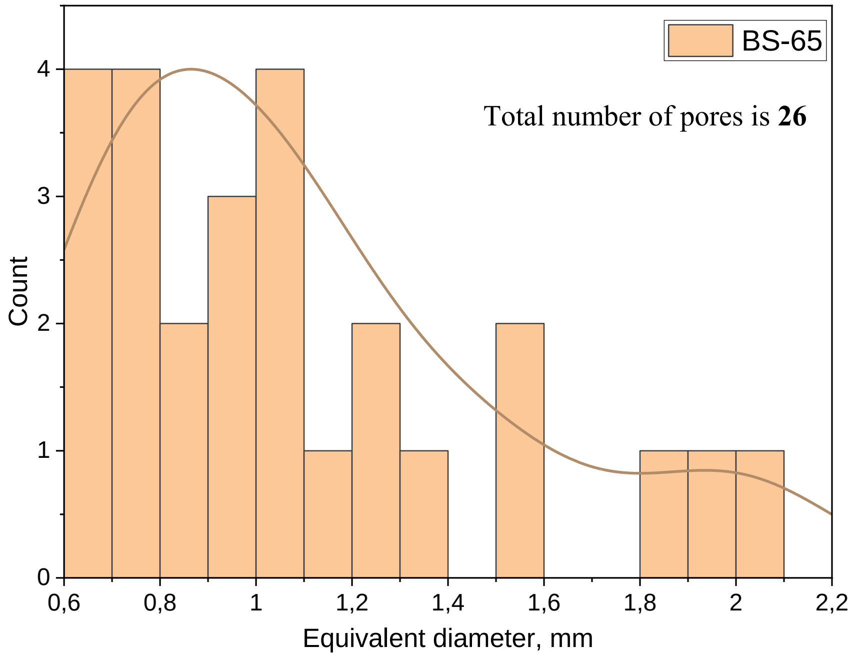

(f) The obtained distributions of the equivalent diameters of the pores with the Kernel smooth distribution curve for sample BS-65.

Figure S2. The obtained distribution of equivalent diameters of pores with the fitted approximation curves using the Kernel Smooth mode.
